# Model for predicting the recurrence of atrial fibrillation after monopolar or bipolar radiofrequency ablation in patients with AF and mitral valve disease

**DOI:** 10.1186/s13019-024-02742-7

**Published:** 2024-05-16

**Authors:** Wei Si, Peng Teng, Liang Ma

**Affiliations:** Department of Cardiovascular Surgery, the First Affiliated Hospital, Zhejiang University School of Medicine, Hangzhou, China

**Keywords:** Atrial fibrillation, Radiofrequency ablation, Recurrence, Predictive model

## Abstract

**Objectives:**

This study aimed to identify the risk factors for postoperative atrial fibrillation in patients with valvular atrial fibrillation, and establish predictive models of atrial fibrillation recurrence.

**Methods:**

Overall, 224 patients who underwent radiofrequency ablation of atrial fibrillation from November 2014 to November 2020 were included. The statistical package for social sciences, X-tile, and R-studio were used for statistical analysis.

**Results:**

Patients were divided into training and validation sets according to a ratio of 3:1. The training set was analysed using univariate and multivariate Cox regression analysis and showed that preoperative uric acid > 401 μmol/L (*P* = 0.006), B-type natriuretic peptide > 202 ng/L (*P* = 0.042), hypersensitivity C-reactive protein > 6.1 mg/L (*P* = 0.026), erythrocyte sedimentation rate > 7.0 mm/h (*P* = 0.016), preoperative left atrial diameter > 48 mm (*P* = 0.031) were significantly correlated with the recurrence of atrial fibrillation after radiofrequency ablation in patients with valvular atrial fibrillation. In the training set, a Cox regression model of the five related factors was established using the R language. The C-index of the model was 0.82, and the area under the receiver operating characteristic curve was 0.831 (*P* < 0.001). Internal and external verification was performed in the training and validation sets, respectively, and both showed that the fit of the verification curve was relatively good at 3 months, 6 months, 1 year, and 3 years postoperatively. After calculating the weight of each related factor using the nomogram, a new risk predictive model (BLUCE) for postoperative atrial fibrillation was established.

**Conclusions:**

In patients with atrial fibrillation, preoperative uric acid, B-type natriuretic peptide, hypersensitivity C-reactive protein, erythrocyte sedimentation rate, and left atrial diameter are risk factors for atrial fibrillation or atrial flutter recurrence after radiofrequency ablation. The BLUCE predictive model can distinguish high-risk groups of postoperative atrial fibrillation. High-risk patients in the BLUCE model were more likely to experience recurrence of atrial fibrillation after radiofrequency ablation and a low possibility of maintaining sinus rhythm.

**Supplementary Information:**

The online version contains supplementary material available at 10.1186/s13019-024-02742-7.

## Introduction

Currently, surgical radiofrequency ablation (MAZE) is one of the most effective treatments for patients with non-isolated atrial fibrillation [1, 2]. The results of long-term follow-up after surgery showed that the proportion of patients maintaining sinus rhythm was 60–85%, and the rest of the follow-up patients had a recurrence of atrial fibrillation or atrial flutter [3, 4].

Relevant studies have pointed out that the recurrence of atrial fibrillation after ablation may be related to age, left atrial diameter, type of atrial fibrillation, postoperative atrial tachycardia, and higher preoperative euroscore. Patients with a left atrial diameter > 70 mm, persistent or permanent atrial fibrillation, or Euro score > 6 points had a higher risk of postoperative recurrence of atrial fibrillation [5–7]. A Chinese multicentre study reported that the recurrence of atrial fibrillation after radiofrequency ablation was associated with right atrial diameter, hypertension, diabetes, and smoking [8]. A new large meta-analysis including more than 50,000 patients from 20 centres examined the association between obesity and the recurrence of atrial fibrillation after catheter ablation. Obese patients (body mass index [BMI] > 28 kg/m^2^) had a higher probability of atrial fibrillation relapse after catheter ablation (OR = 1.30) [9]. The treatment method mentioned in the study was catheter ablation, which was different from the surgical radiofrequency ablation in our study; however, the results could be instructive and inspirational.

In a Japanese multicentre study, 450 patients with non-isolated atrial fibrillation who underwent atrial fibrillation cryoablation maze between 2001 and 2019 were examined. After logistic regression analysis, atrial fibrillation f-wave voltage < 0.2 mV, history of atrial fibrillation > 5 years, and left atrial volume > 100 ml/m^2^ were associated with postoperative atrial fibrillation recurrence. Therefore, a new risk assessment model was established using these three influencing factors, with a maximum score of 10 points. The postoperative recurrence rate of atrial fibrillation was higher in patients with > 7 points [10].

A related paper compared the efficacy of two different surgical methods (modified minimally invasive MAZE using monopolar radiofrequency ablation vs. open surgery MAZE using bipolar radiofrequency ablation) in patients with non-isolated atrial fibrillation [4]. Based on these results, we will further explore the factors related to the recurrence of atrial fibrillation and the maintenance of sinus rhythm after radiofrequency ablation, aiming to quickly and effectively filter patients with higher postoperative atrial fibrillation recurrence risk and select appropriate patients for clinical atrial fibrillation. The treatment plan provides new ideas, and the factors affecting the recurrence of postoperative atrial fibrillation provide valuable references for preventing the recurrence of postoperative atrial fibrillation.

### Patients

This retrospective study included a total of 275 AF patients from two inpatient wards in the First Affiliated Hospital, School of Medicine, Zhejiang University. We divided these patients into two groups——the minimally invasive MAZE group(mi-MAZE group, who underwent modified minimally invasive MAZE with monopolar radiofrequency ablation and mitral valve surgery from January 1, 2014 to November 30, 2020) and the open surgery MAZE group (os-MAZE group, who underwent traditional bipolar radiofrequency ablation and mitral valve surgery from January 1, 2014 to November 30, 2020). Patients with a history of atrial fibrillation less than 2 years and a left atrial diameter < 60 mm with mitral valve disease were selected. 29 patients were excluded because they did not meet the above criteria. 12 patients were excluded due to lack of preoperative echocardiography or laboratory examination. 7 were excluded due to lack of follow-up after surgery, and 1 were excluded due to severe postoperative complications. Eventually 224 AF patients(79 patients in mi-MAZE group and 145 patients in os-MAZE group) were included.

In fact, 107 of the 224 patients had been studied in a previously published article, and we found that there was no statistically significant difference in the postoperative atrial fibrillation recurrence rate (sinus rhythm rate) between mi-MAZE group and os-MAZE group [4]. Therefore, on this basis, we added newly 117 patients from November 2019 to November 2020, and the original conclusion still holds (see Supplementary Materials) that postoperative recurrence of atrial fibrillation in patients with non-isolated atrial fibrillation was not significantly associated with the ablation method received. Therefore, these patients were included in this study. Non-isolated atrial fibrillation patients (224 patients) who underwent radiofrequency ablation of atrial fibrillation from 2014 to 2020 in the Department of Cardiac and Great Vascular Surgery, Affiliated Hospital of Zhejiang University School of Medicine were included in this study.

Preoperative baseline data and intraoperative and postoperative monitoring indicators are summarized in the supplementary materials.

### Surgical technique

The mi-MAZE group: Our hospital used an original surgery technique for the mi-MAZE group—— After general anesthesia, incision was made in the fourth intercostal space of the right chest. After heparinization, peripheral extracorporeal circulation was established through the femoral artery and vein. Blunt dissection and monopolar ablation of the right superior and inferior pulmonary veins were done during extracorporeal circulation. The left upper and lower pulmonary veins are bluntly separated after heart arrest. The Medtronic Cardioblate flushing radiofrequency system which is connected to the monopolar ablation pen was used for ablation. Left atrial ablation path includes: left and right pulmonary vein ring, the line which connects right superior pulmonary vein and left superior pulmonary vein, the line which connects right lower pulmonary vein and left lower pulmonary vein, the line which connects the incision on interatrial groove and mitral annulus, the line which connects left lower pulmonary vein and mitral annulus, and the line which connects left atrial appendage and left superior pulmonary vein. After the ablation is completed, the valve is replaced or repaired and the left atrial appendage is ligated. The epicardial temporary pacing leads are placed routinely.

The os-MAZE group: After general anesthesia, traditional sternotomy was made in the midline. After heparinization, extracorporeal circulation was established through ascending aorta and right atrium. Blunt dissection and bipolar ablation of the right superior and inferior pulmonary veins were done during extracorporeal circulation and the Marshall ligament was cut off. The left upper and lower pulmonary veins are bluntly separated after heart arrest. The Medtronic Cardioblate flushing radiofrequency system which is connected to the bipolar and the mono- polar ablation pen was used for ablation. Left atrial ablation lines were the same as in mi-MAZE group. The right atrium ablation lines include: the line which connects superior vena cava and inferior vena cava, the line which connects right atrial anterior wall incision, coronary sinus and tricuspid posterolateral annulus, the line which connects right atrial anterior wall incision and atrial septal fossa, the line which connects the tricuspid anterior leaflet and the right atrial appendage, and the line which connects the tricuspid posterior valve annulus and the incision on right atrial anterior wall. After the ablation is completed, the valve is replaced or repaired and the left atrial appendage is ligated. The epicardial temporary pacing leads were placed routinely.

The patients’ surgical approaches were determined by the surgeon.

### Follow up

The primary endpoints during the postoperative follow-up were recurrence of atrial fibrillation (or atrial flutter) and death. The main follow-up items were as follows: 3 months after surgery, 6 months after surgery, 1 year after surgery, and at least once a year after the electrocardiography and echocardiography results: electrocardiography to determine whether sinus rhythm was maintained and echocardiography to determine the left atrium anterior and posterior diameter, left atrium upper and lower diameter, left atrium left and right diameter, mitral valve transvalvular pressure difference, left ventricular end-systolic diameter, left ventricular end-diastolic diameter, and left ventricular ejection fraction. Moreover, the medical outcome study (MOS) item short form health survey (SF-36) scores at 3 months, 6 months, and 1 year after surgery, were determined. The follow-up was performed until 15 January 2021.

Since postoperative recurrence of atrial fibrillation in patients with non-isolated atrial fibrillation was not significantly associated with the ablation method received(see Supplementary Materials), we included 224 patients as a whole group to find the reason of postoperative recurrence of non-isolated atrial fibrillation. A total of 224 patients were randomly divided into two groups: the training and validation sets according to the ratio 3:1. Relevant studies have indicated that the ratio of the training and validation sets can vary from 2:1 to 4:1 [1, 2, 11]. The training set data were used for the construction and internal validation of the prediction model, and the validation set data were used for the external validation of the prediction model .

### Statistical analysis

Data were analysed using the SPSS 21.0 software (IBM SPSS Statistics, IBM Corp., Armonk, NY, USA) and Graph Pad Prism(GraphPad Software, San Diego, California, USA). The measurement data that conformed to the normal distribution are represented by the mean and standard deviation, those that did not conform are represented by the median and quartile, and the discrete variables are represented by the frequency and percentage. Measurement data that conformed to the normal distribution were compared using the standard t-test, and those that did not conform were compared using the two-sample Kolmogorov–Smirnov test or the Mann–Whitney test. The difference between the groups of discrete variables was determined using the chi-square test. If the total sample size was ≥40 and the expected frequency in all cells was ≥5, the Pearson chi-square test was used. If the total sample size was ≥40 and there was at least one expected frequency ≤ 5 in the cell, a continuously corrected chi-square test was used. If the total sample size was < 40 or minimum expected frequency < 1, Fisher’s exact test was used. The results were evaluated using a 95% confidence interval and a significance level of *P* < 0.05. Taking the recurrence of atrial fibrillation as the outcome event, the survival curve was drawn using the Kaplan–Meier method, cumulative maintenance sinus rate was evaluated, and difference between groups was tested using the log-rank test.

The continuous variable threshold (cut-off value) associated with the analysis of postoperative atrial fibrillation recurrence was determined by the minimum *P*-value method using X-tile software(Yale university, New Haven, Connecticut, USA) [12]. Univariate and multivariate Cox regression analyses using SPSS software, were used to screen out the statistical factors that were significantly associated with postoperative atrial fibrillation recurrence. Hazard ratios (HR), 95% confidence intervals (CI), and *P* values ​​were evaluated using Cox regression analysis. A prediction model of atrial fibrillation recurrence after radiofrequency ablation was constructed through the screening of relevant factors, and the Cox regression model of relevant factors was established using R language (R studio). The Harrell’s concordance index (C-index) was used to evaluate the prediction model and consistency of the actual situation. The receiver operating characteristic (ROC) analysis and its area under the curve (AUC) were used to evaluate the performance of the model [13]. Internal validation was used to verify the model and judge the degree of bias between the predictive effect of the model and the actual situation. The repeat sampling method was used with a sampling frequency of 1000 [14, 15] to draw a nomogram to obtain the weight of each related factor. The SPSS software was used for survival analysis and the Kaplan–Meier method was used to draw the survival curve with postoperative atrial fibrillation recurrence as the outcome event, compare the differences between the subgroups, and establish a simplified atrial fibrillation recurrence prediction scale. Cox univariate regression was used to analyse the differences between groups of the scale, and external validation was used to verify the scale model through the validation set. The repeated sampling method was also used with a sampling frequency of 1000. The C-index was used to evaluate the consistency of the predicted situation with the actual situation.

The SPSS software was used for statistical analysis, X-tile 3.6.1 software to determine continuous variable threshold, and R 4.0.3 and R studio software(RStudio Inc., JJ Allaire, Boston, Massachusetts, USA) for R language programming. The R language software package uses rms, Hmisc, Survival, open, openxlsx, lattice, Formula, and ggplot2 for package implementation. Statistical significance was set at *P* < 0.05.

## Result

The preoperative baseline characteristics of the patients included in the study are shown in Table S1. Thirty three men(41.8%) and 46 women(58.2%) were included in the mi-MAZE group, and the os-MAZE group included 51 men and 95 women(*P* = 0.148). The type of atrial fibrillation in both groups was concentrated in paroxysmal (72.2% in the mi-MAZE group and 70.5% in the os-MAZE group), the proportion of persistent atrial fibrillation was smaller (27.8% vs 29.5%), and there was no permanent atrial fibrillation in either group.

Characteristics and events during the operation were included in Table S2. Two groups who underwent radiofrequency ablation also underwent other heart valve surgery. Since all patients were suffering from mitral valve disease, they all underwent mitral valve replacement or plastic surgery. There was no significant difference in overall operation time between the two groups (*P* = 0.472).

After surgery, we followed up 244 patients of two groups (1 patient of os-MAZE group were excluded due to severe postoperative complications, Table 1). It was found that 65 patients (82.29%) in the mi- MAZE group and 109 patients (75.17%) in the os-MAZE group were sinus rhythm 3 months after operation(*P* = 0.244). Similar results were also found 6 months and 12 months after surgery. Therefore, we could say that the surgical methods do not affect the recurrence probability.

**Table 1:** Echocardiographic and ECG data between the two groups 3, 6, and 12 months after operation

	mi-MAZE^a^	os-MAZE^b^	*P* value
3 months after sugery			
Patients	79	145	
Sinus rhythm	65(82.29%)	109(75.17%)	0.244
LAD(mm)	39.0 ± 9.00	40.0 ± 8.00	0.168
LASID(mm)	69.0 ± 11.9	63.8 ± 9.66	0.001
LAMD(mm)	59.0 ± 16.0	56.0 ± 13.0	0.069
MVG(mmHg)	5.20 ± 4.10	5.60 ± 2.80	0.107
LVDs(mm)	34.0 ± 8.00	36.0 ± 5.00	0.010
LVDd(mm)	49.8 ± 6.33	49.4 ± 5.63	0.807
LVEF(%)	62.5 ± 6.57	63.8 ± 5.53	0.167
SF-36(points)	120.61 ± 8.53	110.21 ± 6.90	< 0.001
6 months after sugery			
Patients	73	134	
Sinus rhythm	56(76.71%)	96(71.64%)	0.511
LAD(mm)	38.2 ± 5.31	39.7 ± 7.42	0.161
LASID(mm)	65.0 ± 14.0	65.0 ± 13.0	0.645
LAMD(mm)	55.0 ± 11.0	56.0 ± 13.0	0.127
MVG(mmHg)	4.20 ± 3.00	3.70 ± 3.00	0.083
LVDs(mm)	34.0 ± 6.00	36.0 ± 5.00	0.040
LVDd(mm)	50.6 ± 6.84	51.6 ± 6.04	0.248
LVEF(%)	65.0 ± 8.00	64.0 ± 7.50	0.416
SF-36(points)	121.20 ± 7.91	112.18 ± 6.17	< 0.001
12 months after sugery			
Patients	61	118	
Sinus rhythm	44(72.13%)	79(66.95%)	0.478
LAD(mm)	40.6 ± 7.24	40.1 ± 7.68	0.771
LASID(mm)	61.0 ± 17.5	62.5 ± 11.3	0.356
LAMD(mm)	53.4 ± 11.4	55.0 ± 14.0	0.334
MVG(mmHg)	4.10 ± 2.95	3.00 ± 3.25	< 0.001
LVDs(mm)	33.0 ± 5.50	33.0 ± 4.00	0.140
LVDd(mm)	50.7 ± 6.34	50.0 ± 7.00	0.224
LVEF(%)	64.7 ± 5.73	64.0 ± 7.00	0.229
SF-36(points)	122.80 ± 7.19	118.40 ± 6.55	< 0.001

^a^mi-MAZE: modified minimally invasive MAZE using monopolar radiofrequency ablation; ^b^ os-MAZE: open surgery MAZE using bipolar radiofrequency ablation

**Fig. 1:**
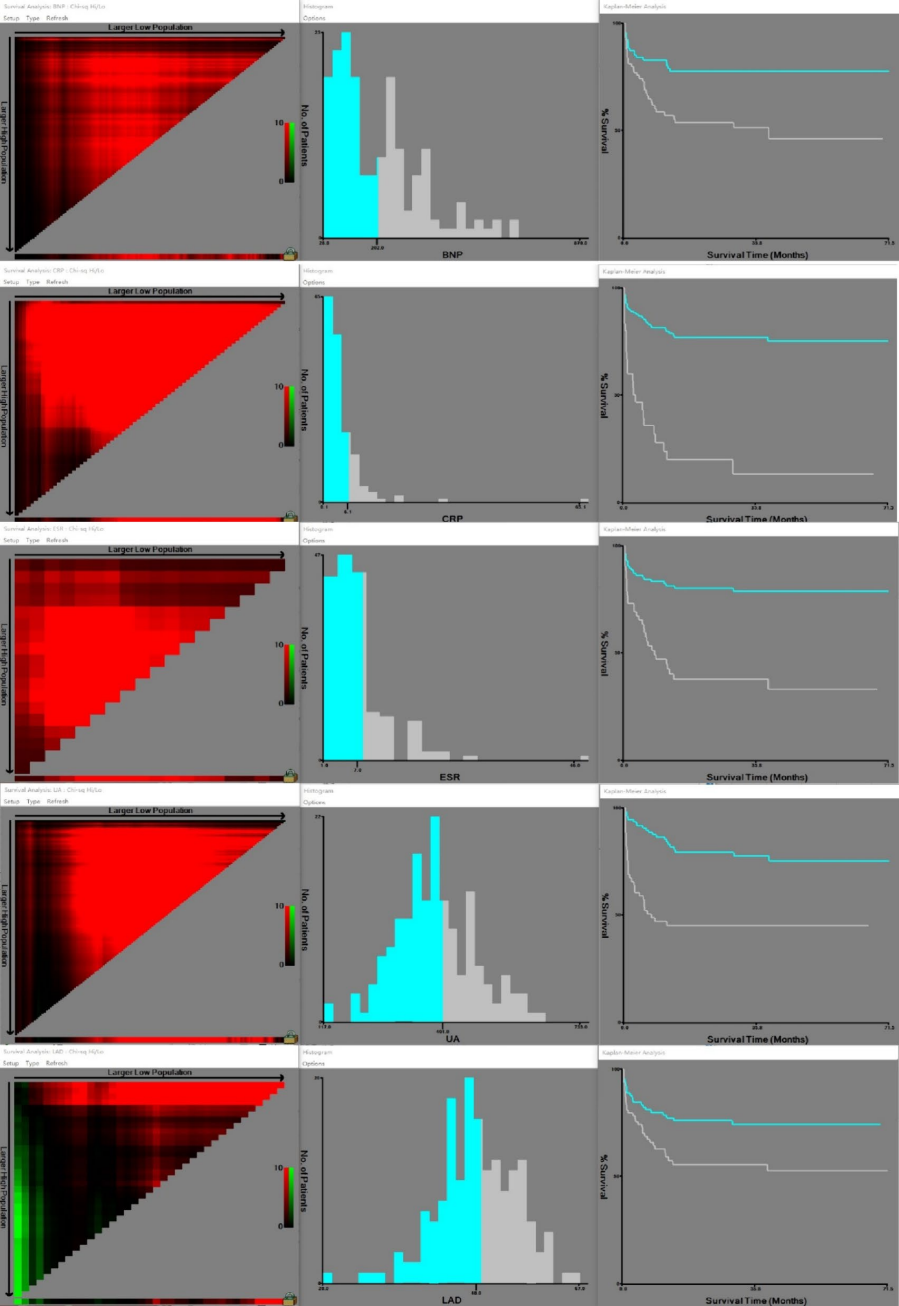
Thresholds determined by X-tile

To screen out the risk factors for the recurrence of atrial fibrillation in patients with non-isolated atrial fibrillation after radiofrequency ablation, we used segmentation of continuous variables, univariate and multivariate Cox regression analyses to analyse the sex, age, cardiac function, type of atrial fibrillation, complications, preoperative test indicators and echocardiographic data of 168 patients in the training set, and a new scoring tool for predicting the recurrence of atrial fibrillation after radiofrequency ablation was constructed. Continuous variables were segmented using the X-tile to determine thresholds (Fig. 1 and Fig. S2).

Individual categorical and continuous variables in the training set were analysed using one-way Cox regression according to the thresholds for each continuous variable obtained by the X-tile (Table 2).

**Table 2:** Training set:Univariate Cox regression analysis

Category	n	Recurrence(%)	HR(95%CI)	*P* value
Sex				
Male	66	22(33.33%)	1	
Female	102	33(32.35%)	0.952(0.555–1.632)	0.857
Age(y)				
≤68	140	48(34.29%)	1	
≥69	28	7(25.00%)	0.707(0.320–1.562)	0.391
NYHA				0.890
II	105	33(31.43%)	1	
III	41	14(34.15%)	1.088(0.582–2.033)	0.793
IV	22	8(36.36%)	1.197(0.553–2.593)	0.648
Atrial fibrillation type				
Paroxysmal	119	37(31.09%)	1	
Persistent	49	18(36.73%)	1.289 (0.733–2.265)	0.378
Hypertension				
Yes	113	39(34.51%)	1	
No	55	16(29.09%)	0.891(0.498–1.595)	0.698
Palpitations				
Yes	99	33(33.33%)	1	
No	69	22(31.88%)	0.954(0.556–1.636)	0.864
Pulmonary arterial hypertension				
Yes	115	31(26.96%)	1	
No	53	24(45.28%)	2.007(1.174–3.430)	0.011
Uric acid(μmol/L)				
≤401	107	22(20.56%)	1	
> 401	61	33(54.10%)	3.604(2.097–6.195)	< 0.001
B-type natriuretic peptide(ng/L)				
≤202	94	20(21.28%)	1	
> 202	74	35(47.30%)	2.537(1.462–4.404)	0.001
High-sensitivity C-reactive protein(mg/L)				
≤6.1	137	31(22.63%)	1	
> 6.1	31	24(77.42%)	5.591(3.244–9.636)	< 0.001
Erythrocyte sedimentation rate(mm/h)				
≤7.0	116	23(19.83%)	1	
> 7.0	52	32(61.54%)	3.992(2.329–6.841)	< 0.001
Platelet(×10^^^9/L)				
≤257	137	38(27.74%)	1	
> 257	31	17(54.84%)	2.315(1.306–4.106)	0.004
D-dimer(μg/L)				
≤562	117	30(25.64%)	1	
> 562	51	25(49.02%)	2.199(1.292–3.744)	0.004
Left atrial diameter(mm)				
≤48	90	22(24.44%)	1	
> 48	78	33(42.31%)	2.029(1.181–3.486)	0.010
Left ventricular end-systolic dimension(mm)				
≤31	64	26(40.63%)	1	
> 31	104	29(27.88%)	0.620(0.365–1.053)	0.077
Left ventricular end-diastolic dimension(mm)				
≤42	17	8(47.06%)	1	
> 42	151	47(31.13%)	0.576(0.272–1.221)	0.150
Left ventricular ejective fraction(%)				
≤60	73	20(27.40%)	1	
> 60	95	35(36.84%)	1.439(0.831–2.493)	0.194
Mitral transmitral pressure gradient(mmHg)				
≤8.2	139	33(23.74%)	1	
> 8.2	29	22(75.86%)	4.856(2.800–8.421)	< 0.001
Left atrial superior and inferior diameter(mm)				
≤78	125	32(25.60%)	1	
> 78	43	23(53.49%)	2.358(1.378–4.032)	0.002
Left atrial left and right diameter(mm)				
≤75	147	38(25.85%)	1	
> 75	21	17(80.95%)	4.680(2.618–8.367)	< 0.001

Univariate Cox regression analysis showed that pulmonary hypertension (*P* = 0.011), preoperative uric acid (UA) level (*P* < 0.001), preoperative B-type natriuretic peptide (BNP)(*P* = 0.001), preoperative high-sensitivity C-reactive protein (CRP) (*P* < 0.001), preoperative erythrocyte sedimentation rate (ESR) (*P* < 0.001), preoperative platelet count (*P* = 0.004), preoperative D-dimer level (*P* = 0.004), preoperative left atrial diameter (LAD) (*P* = 0.010), preoperative MVG (*P* < 0.001), preoperative LASID (*P* = 0.002), and preoperative LAMD (P < 0.001) were significantly correlated with the recurrence of atrial fibrillation after radiofrequency ablation. Multivariate Cox regression analysis (Table 3) was performed for these variables.

**Table 3:** Training set: Multivariate Cox regression analysis

Category	HR(95%CI)	*P* value
Pulmonary arterial hypertension		
Yes	1	
No	1.151(0.623–2.126)	0.654
Uric acid(μmol/L)		
≤ 401	1	
> 401	2.319(1.279–4.208)	0.006
B-type natriuretic peptide(ng/L)		
≤ 202	1	
> 202	1.852(1.023–3.353)	0.042
high-sensitivity C-reactive protein(mg/L)		
≤ 6.1	1	
> 6.1	2.220(1.101–4.478)	0.026
Erythrocyte sedimentation rate(mm/h)		
≤ 7.0	1	
> 7.0	2.144(1.152–3.988)	0.016
Platelet(×10^^^9/L)		
≤ 257	1	
> 257	1.298(0.628–2.683)	0.481
D-dimer(μg/L)		
≤ 562	1	
> 562	1.584(0.871–2.880)	0.132
Left atrial diameter(mm)		
≤ 48	1	
> 48	1.936(1.063–3.523)	0.031
Mitral transmitral pressure gradient(mmHg)		
≤ 8.2	1	
> 8.2	2.273(1.227–4.210)	0.009
Left atrial superior and inferior diameter(mm)		
≤ 78	1	
> 78	0.782(0.288–2.121)	0.629
Left atrial left and right diameter(mm)		
≤ 75	1	
> 75	2.911(1.027–8.246)	0.044

According to multivariate Cox regression analysis, preoperative UA (*P* = 0.006), BNP (*P* = 0.042), high-sensitivity CRP (*P* = 0.026), ESR (*P* = 0.026) =0.016), LAD (*P* = 0.031), MVG (*P* = 0.009), and LAMD (*P* = 0.044) were significantly associated with atrial fibrillation recurrence after radiofrequency ablation in patients with non-isolated atrial fibrillation.

Univariate and multivariate Cox regression analyses showed that preoperative UA, BNP, high-sensitivity CRP, ESR, LAD, MVG, and LAMD were significantly associated with radiofrequency ablation in patients with non-isolated atrial fibrillation. The recurrence of atrial fibrillation was statistically significant; however, when establishing a predictive model for the recurrence of atrial fibrillation, in addition to considering statistical significance, it was necessary to consider whether these indicators are clinically relevant. Since we could predict the recurrence of atrial fibrillation after surgery, we could determine predictors related to atrial myocardium inflammation or atrial fibrosis. Therefore, UA, BNP, five indicators of high-sensitivity CRP, ESR, and LAD were used to establish a predictive model for postoperative atrial fibrillation recurrence. Taking postoperative atrial fibrillation recurrence as the outcome event, five variables were divided into binary variables by X-tile segmentation, and the Cox regression model between the five variables and the outcome event was established using the R language software, namely, a risk prediction model for the recurrence of atrial fibrillation after radiofrequency ablation (Supplementary materials: Code of R language).

**Fig. 2:**
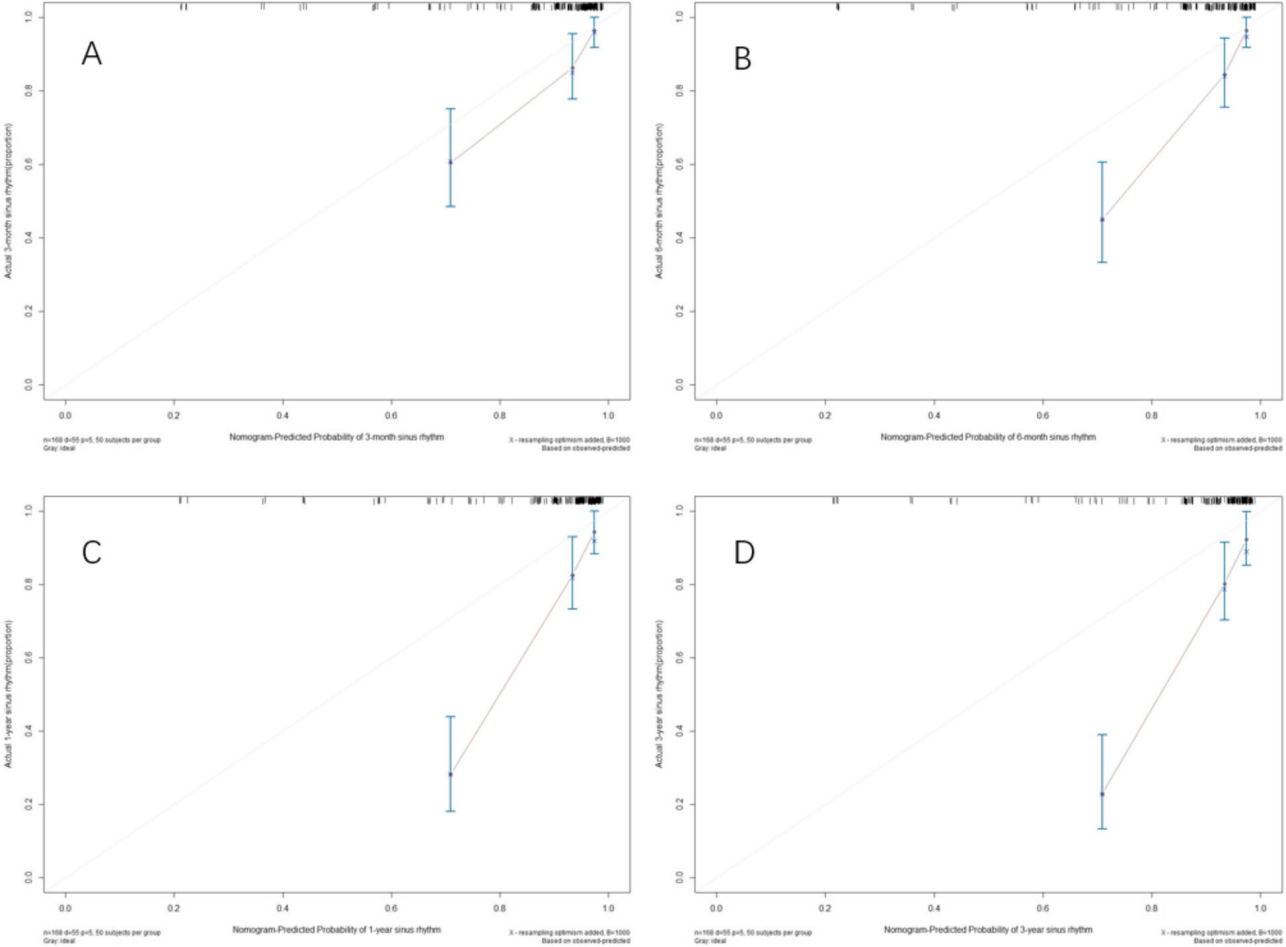
Training set: internal verification. The abscissa is the predicted sinus rate, and the ordinate is the actual postoperative maintenance sinus rate, “·” is the actual maintenance sinus rate of each subgroup, “×” is the corrected maintenance sinus rate obtained by repeated sampling 1000 times, the diagonal line is the reference line, The vertical bar is the 95% confidence interval. **A**: 3 months; **B**: 6 months; **C**: 1 year; **D**:3 years after surgery

The C value of the prediction model was 0.82, indicating that the predicted value had a high degree of fit with the actual value. To further verify the consistency between the predicted value and the actual value, we conducted internal verification of this model to determine whether the model predicts atrial fibrillation. The effect of recurrence and the degree of bias in the real situation were selected as the outcome events at 3 months, 6 months, 1 year, and 3 years after radiofrequency ablation. Internal validation was performed on the training set population and an internal validation calibration chart was drawn (Fig. 2).

The internal validation curve showed that the maintained sinus rate predicted by the model had a high degree of fit and agreement with the actual value at 3 months postoperatively, and the predicted value was within the 95% confidence interval of the actual value. One subgroup had predicted values ​​outside the 95% confidence interval at 3 months, 6 months, 1 year, and 3 years; however, the other two subgroups remained within the confidence interval. Furthermore, the reference C value was 0.82, indicating that the model can better predict the actual maintenance sinus rate.

**Fig. 3:**
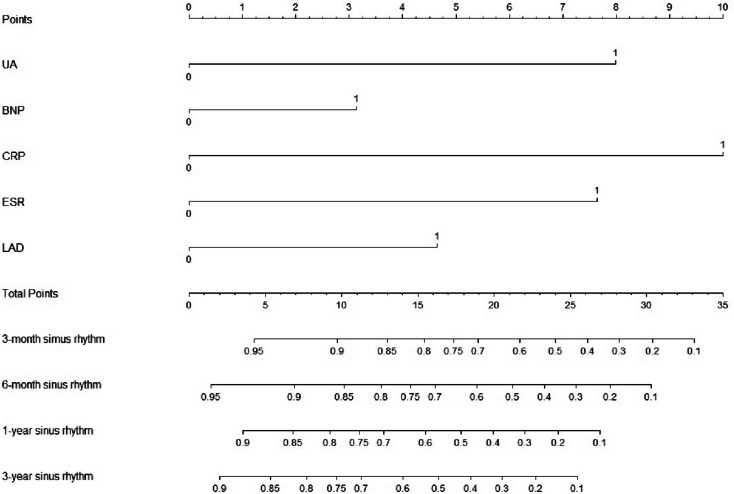
Training set: Nomogram

According to the prediction model, R language was used to draw a nomogram related to five variables, and the weight of each influencing factor was calculated according to the nomogram. The total score was 10 points, and the higher the score, the greater the weight (Fig. 3).

According to the nomogram and weighted score of each influencing factor, we can obtain a new tool for predicting the recurrence of atrial fibrillation after radiofrequency ablation and using it to re-score the 168 patients in the training set, a total of 0 points to 33.41 points can be obtained. There were 25 or 32 scores within this range, and the recurrence of atrial fibrillation was used as the outcome event. Kaplan–Meier analysis was used to construct the survival curve. The distribution of the 32 curves was relatively discrete and could not be used in clinical applications due to a high risk of recurrence. To quickly and effectively classify patients with atrial fibrillation in clinical practice and facilitate the prediction of postoperative recurrence, the prediction model was re-simplified and used. There were three classification methods used (Tables S3/S4/S5).

**Fig. 4:**
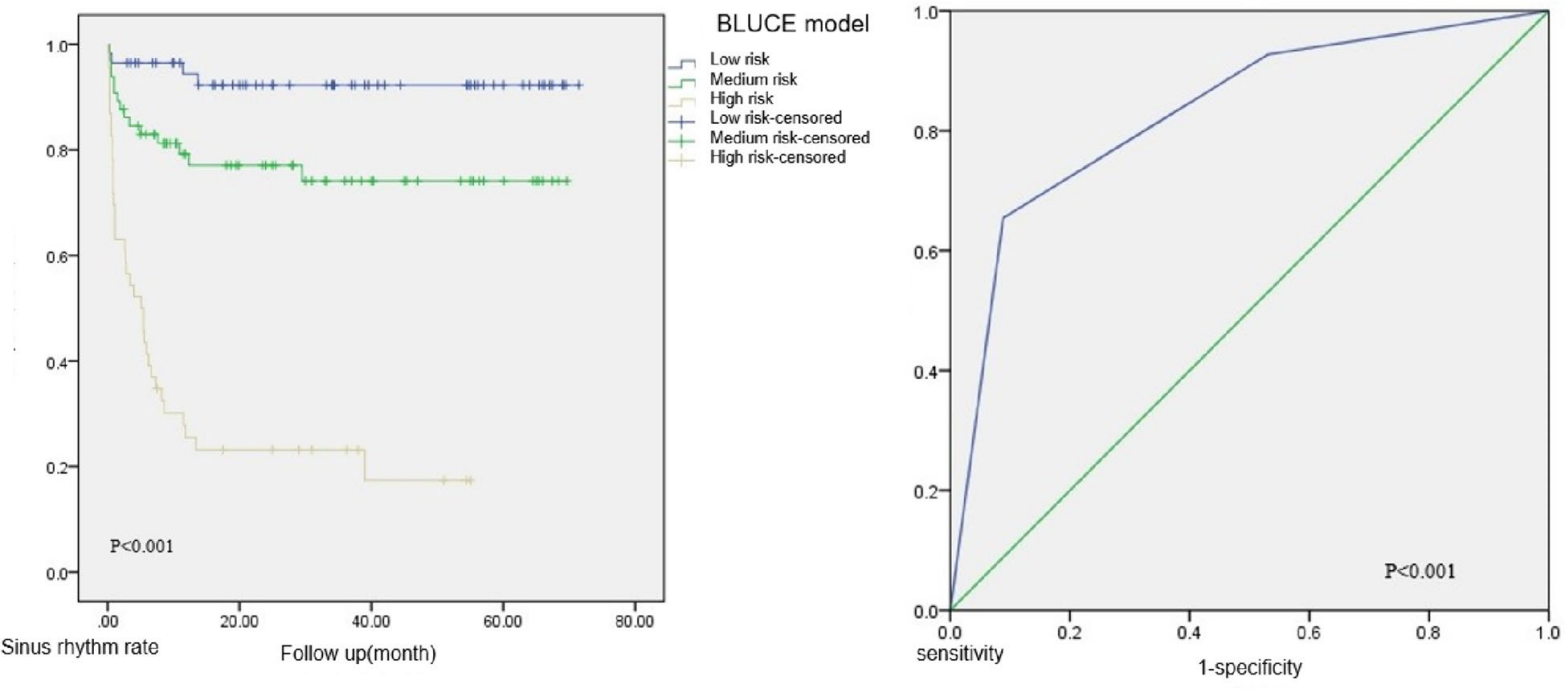
Training set: the Kaplan-Meier estimates and area under ROC curve of BLUCE model. AUC = 0.831(*P* < 0.001)

The Prediction Model Simplified Scale C is adjusted on the basis of B, with 0–1 grades as low risk, 2–3 grades as medium risk, and 4–5 grades as high risk. The Kaplan–Meier survival curve showed a log-rank test obtained (*P* < 0.001). There was a significant statistical difference between the three groups, the sample size distribution of the three groups was relatively balanced, and the follow-up time was relatively similar, which is suitable for predicting the risk of recurrence of atrial fibrillation (Fig. 4). The effectiveness of the simplified scale 3 prediction model in the training set population was tested again and a single-factor Cox regression analysis was used. The results showed that the simplified scale 3 predictive model was significantly correlated with the recurrence of atrial fibrillation after radiofrequency ablation (*P* < 0.001) (Table 4). Therefore, we referred to the simplified model 3 as the “BLUCE” scale - BNP-LAD-UA-CRP-ESR. In ROC analysis, the AUC of the BLUCE prediction model was 0.831 (P < 0.001) with the postoperative atrial fibrillation recurrence as the outcome events (Fig. 4).

**Table 4:** Training set: univariate Cox regression analysis for BLUCE

Grade	n	Recurrence(%)	HR(95%CI)	*P* value
BLUCE model				< 0.001
Low risk	57	4(7.02%)	1	
Medium risk	65	15(23.08%)	3.783(1.255–11.406)	0.018
High risk	46	36(78.26%)	19.196(6.790–54.266)	< 0.001

**Fig. 5:**
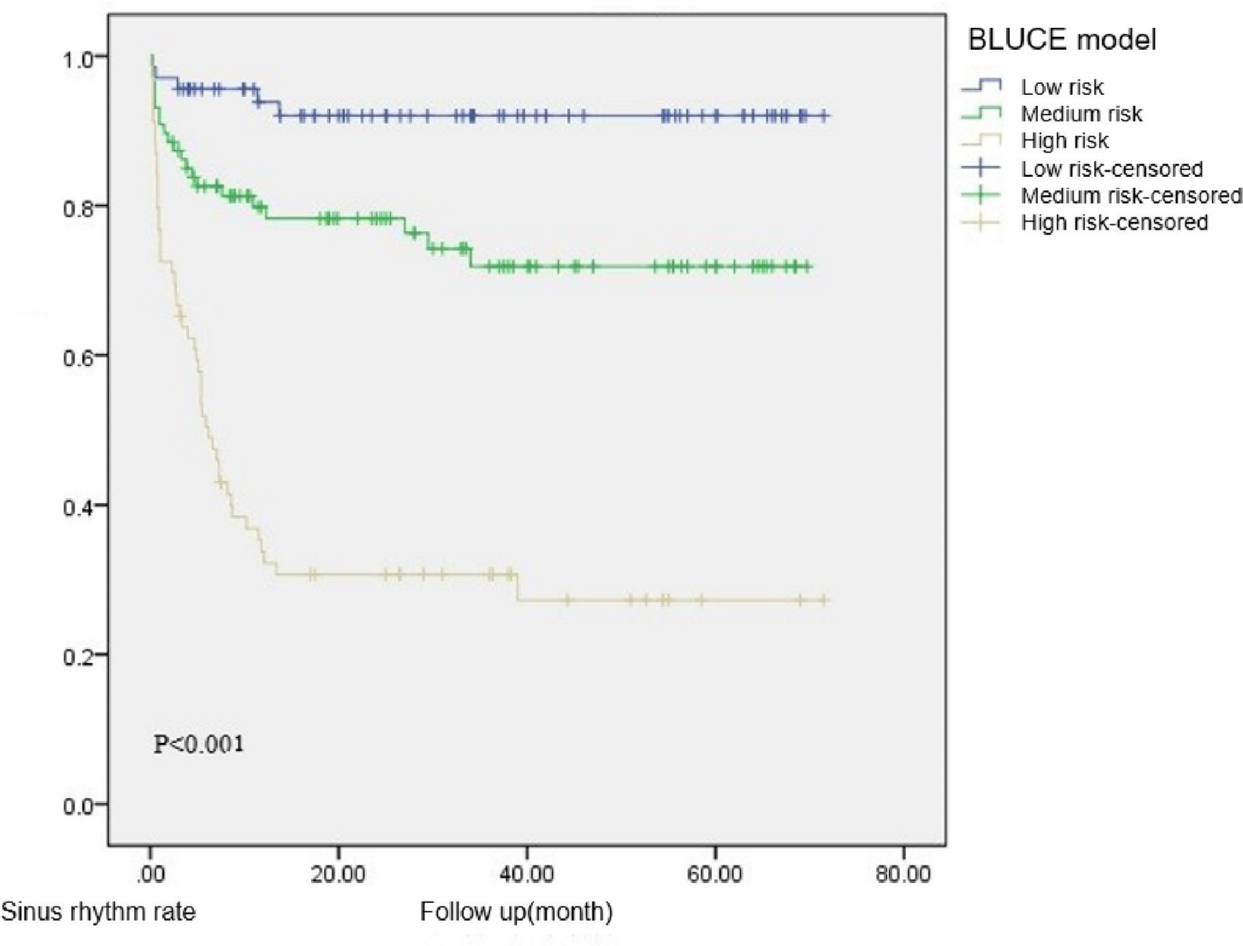
General population: the Kaplan-Meier estimates of BLUCE model

The BLUCE prediction model was applied to 56 patients in the validation set and the general population, and the BLUCE scores and risk grading of all patients in the validation set were calculated. The recurrence of postoperative atrial fibrillation was used as the outcome, and survival curves were plotted using the Kaplan–Meier method. The log-rank test was used for *P*-values (Fig. 5 and Fig. S3).

The efficacy of the BLUCE prediction model in the total population and validation set population was tested using the univariate Cox regression analysis. The results showed that the BLUCE prediction model was significantly correlated with the recurrence of atrial fibrillation after radiofrequency ablation in both the total population and the validation set population (*P* < 0.001 for the total population and *P* = 0.044 for the validation set population) (Table 5 and Table S6). In addition, we used external verification to judge the degree of bias between the effect of the BLUCE prediction model in predicting the recurrence of atrial fibrillation and the actual situation (Fig. S4), which showed that the BLUCE prediction model can be extended to other populations and has relatively good clinical prediction efficacy.

**Table 5:** General population: univariate Cox regression analysis for BLUCE

Grade	n	Recurrence(%)	HR(95%CI)	*P* value
BLUCE model				< 0.001
Low risk	68	5(7.35%)	1	
Medium risk	87	21(24.14%)	3.711(1.399–9.843)	0.008
High risk	69	48(69.57%)	14.429(5.728–36.347)	< 0.001

## Discussion

The recurrence of atrial fibrillation after radiofrequency ablation is associated with many factors. In this study, the recurrence of atrial fibrillation and the maintenance of sinus rhythm after ablation were related to UA, BNP, platelets count, ESR, and LAD. The postoperative atrial fibrillation recurrence prediction model, BLUCE, established by the above influencing factors was divided into three grades: low-risk, intermediate-risk, and high-risk according to the different scores. The higher the grade, the greater the risk of postoperative recurrence of atrial fibrillation.

UA is the final product of the purine metabolism in higher animals. Under physiological regulation, the synthesis and excretion of uric acid in the body maintain a dynamic balance, however, imbalance leads to hyperuricaemia. The mechanism by which elevated UA levels promote atrial fibrillation and thrombosis is unclear, and one possible explanation is that the high levels of UA in vivo induce oxidative stress and inflammation. Hyperuricaemia is independently associated with increased left atrial diameter [16], which is closely related to atrial fibrillation and left atrial appendage thrombosis. BNP and N-terminal pro-brain natriuretic peptide (NT-proBNP) can be used as biomarkers for cardioembolism aetiology in cryptogenic stroke. In a study of 320 patients over 55 years of age with cryptogenic stroke, 22.9% developed atrial fibrillation at the 28-day follow-up [17]. In another meta-analysis that included nearly 30,000 people, in patients with atrial fibrillation, elevated BNP and NT-proBNP levels were significantly associated with adverse outcomes, including mortality (RR = 1.92) and stroke (RR = 1.92) = 2.53), indicating that the level of BNP/NT-proBNP plays an important role in the risk stratification of the prognosis of patients with atrial fibrillation [18].

High-sensitivity CRP level is believed to be associated with both the maintenance and recurrence of atrial fibrillation. Prospective studies in some Western countries have shown that increased expression of serum high-sensitivity CRP can be used as a marker of systemic inflammation and as a risk factor for the development of atrial fibrillation [19].

The relationship between the ESR and the occurrence and maintenance of atrial fibrillation is unclear, and there are few relevant studies. In a study on cardiac myxoma with atrial fibrillation, 51% of patients had an elevated ESR [20].

Many studies have reported the effects of left atrial diameter or volume on the development and maintenance of atrial fibrillation. Inpatients with mitral valve and atrial fibrillation, after surgical radiofrequency ablation, the recurrence rate of atrial fibrillation (maintenance of sinus rhythm) was significantly correlated with the preoperative left atrial diameter [21].

This study had some limitations. First, approximately 6 years of data were collected; however, the overall sample size was still small, especially the sample size of the validation set, which may lead to certain statistical errors in the statistical and validation results of the prediction model. BMI, hypertension, and other related factors were not significantly correlated with the recurrence of atrial fibrillation in this study. We will continue to collect data on patients with non-isolated atrial fibrillation to expand the sample size and reduce the statistical sample bias. Second, the collection of preoperative-related information is not perfect, and there may be other indicators that were not collected but are related to the recurrence of atrial fibrillation, such as angiotensin converting enzyme 2(ACE2) or blood mononuclear cells/high-density lipoprotein. More comprehensive studies on the risk factors associated with recurrent atrial fibrillation after radiofrequency ablation with improved collection of preoperative-related information are needed.

The novelty of this study is that, for the first time, five related factors including the levels of preoperative uric acid, BNP, CRP, ESR, and LAD were combined to establish a new and effective postoperative atrial fibrillation recurrence prediction model that can quickly identify radiofrequency in clinical practice. The utilization of these parameters can provide new ideas to the diagnosis and treatment of atrial fibrillation in the future, such as whether preoperative interventions can reduce the levels of uric acid, BNP, CRP, ESR, and LAD in patients, improve the maintenance rate of sinus rhythm after radiofrequency ablation, and reduce the recurrence rate of atrial fibrillation among high-risk patients.

## Conclusion

Preoperative UA, BNP, high-sensitivity CRP, ESR, and LAD in patients with non-isolated atrial fibrillation were significantly correlated with the recurrence of atrial fibrillation after radiofrequency ablation. Based on the above five related parameters, we established a new model for predicting the risk of postoperative atrial fibrillation(or atrial flutter) recurrence, BLUCE, and calculated the score according to the weight of the five related parameters. The higher the score, the greater the risk of recurrent atrial fibrillation after radiofrequency ablation and the lower the proportion of maintaining sinus rhythm.

### Supplementary Information

**Supplementary Material 1.**

## Data Availability

No datasets were generated or analysed during the current study.

## Abbreviations

CI: Confidence Interval
C-index: Concordance index
LAD: Left atrial diameter
ESR: Erythrocyte sedimentation rate
BNP: B-type natriuretic peptide
UA: Uric acid
NT-proBNP: N-terminal pro-brain natriuretic peptide
CRP: C-reactive protein
MVG: mitral transmitral pressure gradient
LASID: left atrial superior and inferior diameter
LAMD: left atrial left and right diameter
