# Supplementary material for: Model for predicting the recurrence of atrial fibrillation after monopolar or bipolar radiofrequency ablation in patients with AF and mitral valve disease
Source: J Cardiothorac Surg. 2024 May 16;19:290. doi: 10.1186/s13019-024-02742-7 (PMC11097403; doi:10.1186/s13019-024-02742-7)
Supplement: Supplementary file 1 — Supplementary Material 1. [file 13019_2024_2742_MOESM1_ESM.pdf]

Supplementary materials

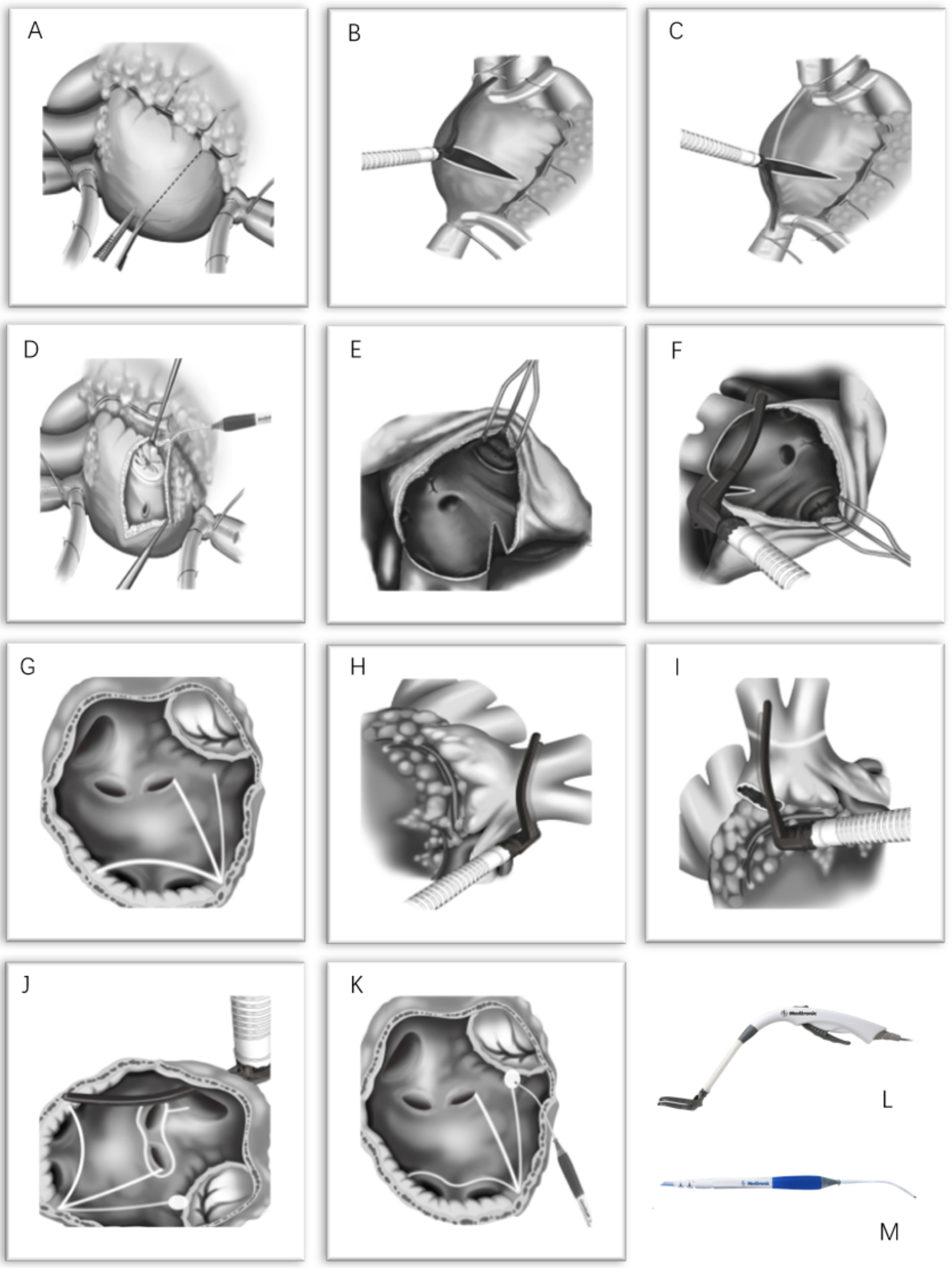

Figure S1: Diagram of the open surgery MAZE using bipolar radiofrequency ablation.

A. Right atrium free wall incision; B. Superior vena cava ablation; C. Inferior vena cava

ablation; D. Anterior tricuspid valve annulus ablation; E. Room sulcus incision; F. vestibular isolation of right pulmonary vein; G. ablation of left atrium bottom and isthmus; H. vestibular isolation of left pulmonary vein; I. ablation of the connection between left atrial appendage and left superior pulmonary vein ostium; J. ablation of left atrium top; K. unipolar Pen supplemental ablation; L. bipolar forceps; M. monopolar pen

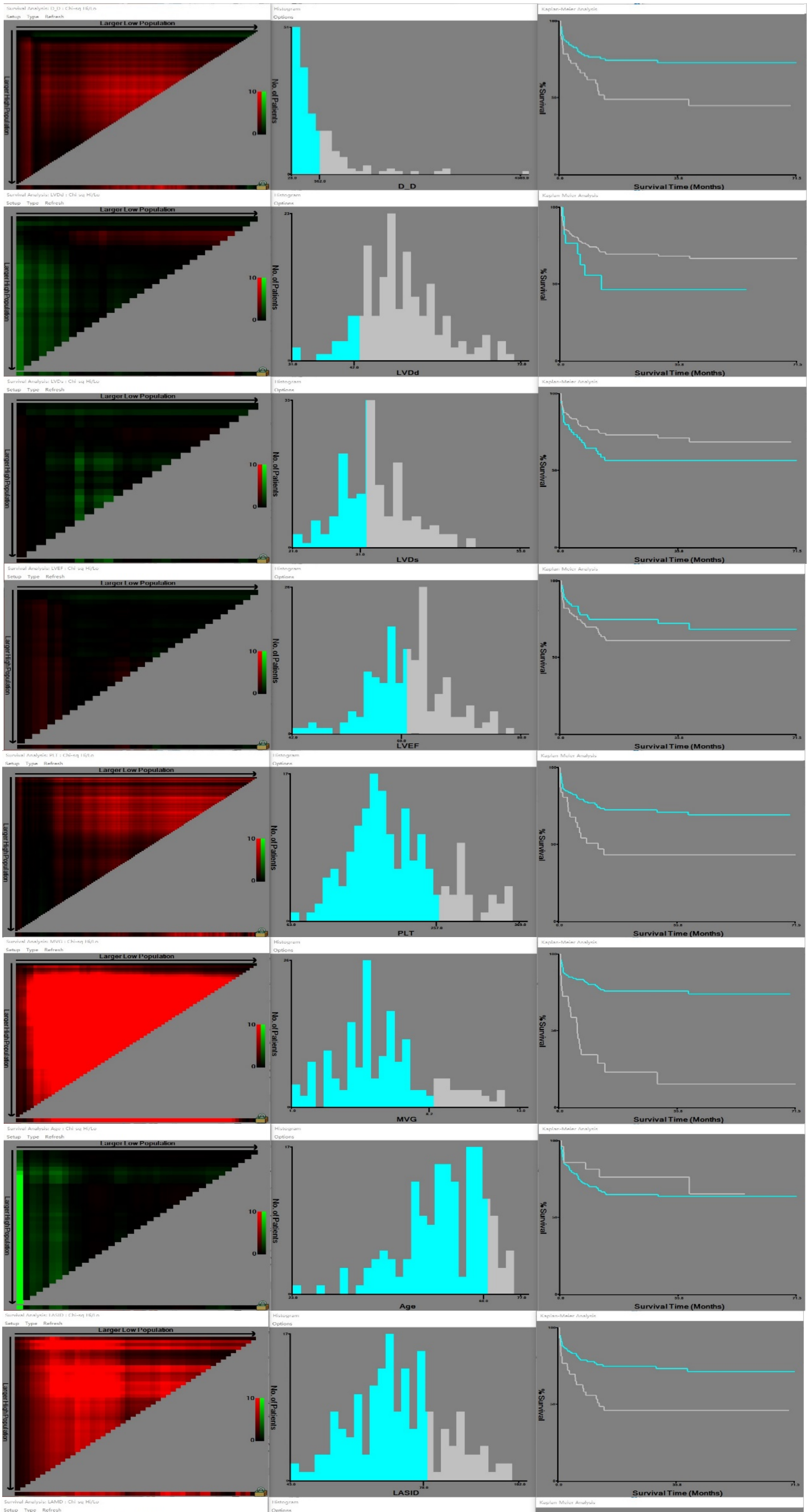

Larger Low Population
Larger High Population
No. of Patients
D_D
% Survival
Survival Time (Months)
LVDd
LVDs
LVEF
PLT
MVG
Age
LASID

Figure S2: Thresholds determined by X-tile. D_D: preoperative D-dimer, cut-off =562.0 μg/L; LVDd: preoperative left ventricular end-diastolic dimension, cut-off =42.0 mm; LVDs: preoperative left ventricular end-systolic dimension, cut-off =31.0 mm; LVEF: preoperative left ventricular ejective fraction, cut-off =60.0%; PLT: preoperative platelet; MVG: preoperative mitral transmitral pressure gradient, cut-off =8.2 mmHg; Age: cut-off =68y; LASID: preoperative left atrial superior and inferior diameter, cut-off =78.0 mm; LAMD: preoperative left atrial left and right diameter, cut-off =75.0mm.

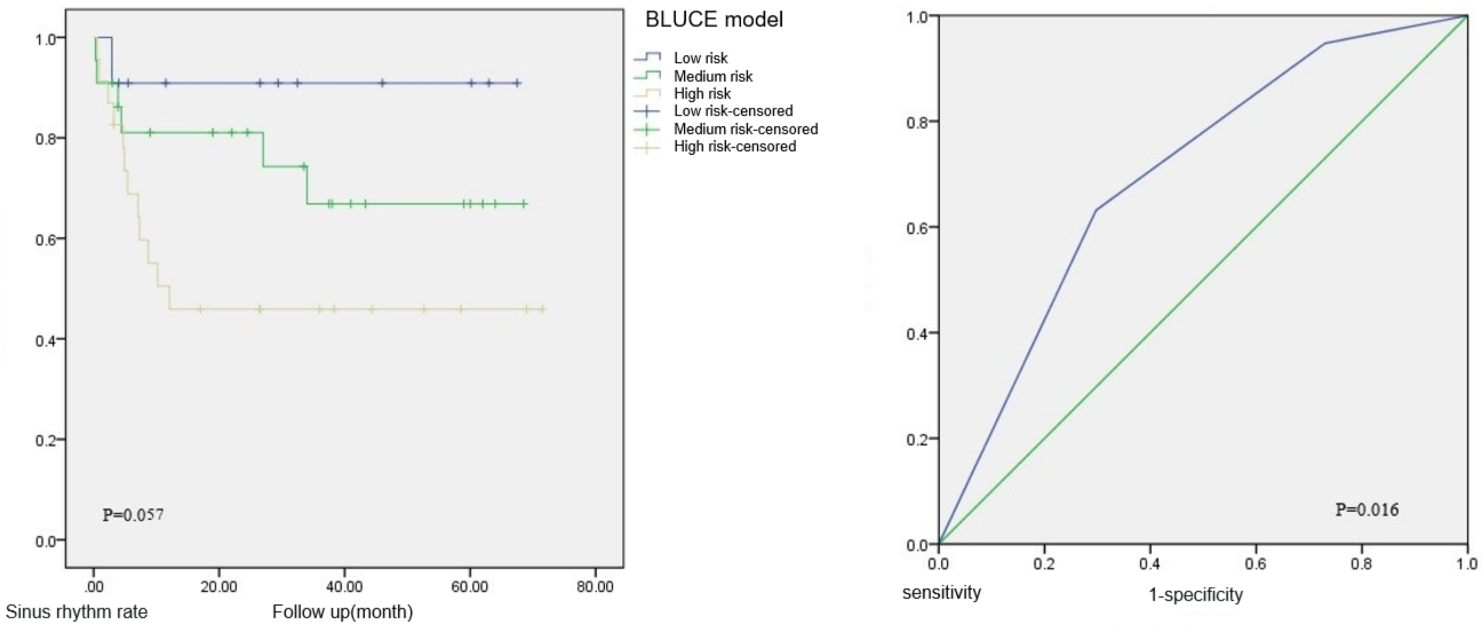

Figure S3: Validation set: the Kaplan-Meier estimates and area under ROC curve of BLUCE model. AUC = 0.698(P=0.016)

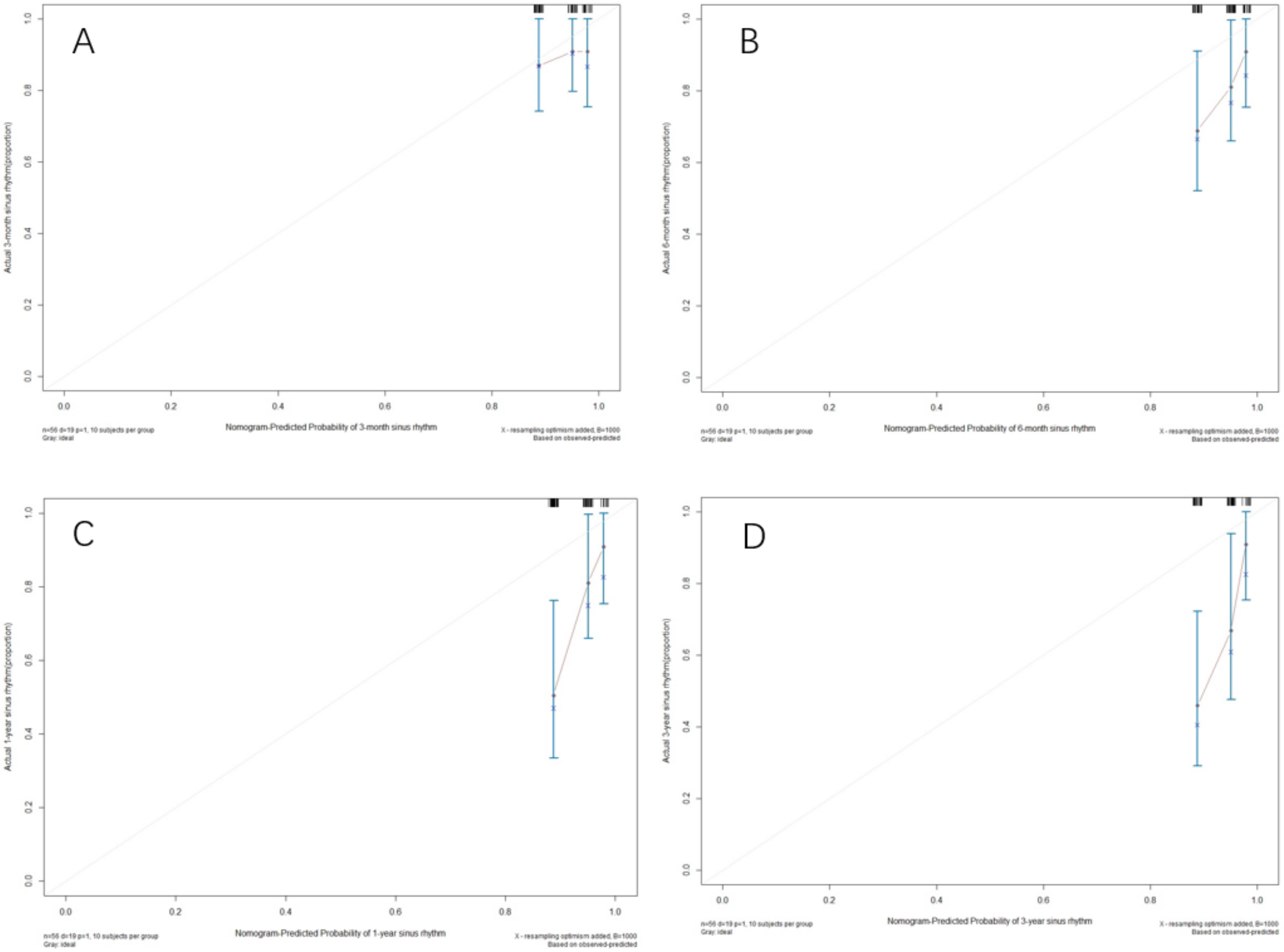

Figure S4: Validation set: external verification.

A: 3 months; B: 6 months; C: 1 year; D:3 years after surgery.

Table S1: The preoperative baseline characteristics of the patients

	mi-MAZE	os-MAZE	*P value*
Number	79	146	-
Gender, F/M（%）	33/46（41.8%/58.2%）	51/95（34.9%/65.1%）	0.317
Age, mean±SD	59.0±14.0	60.0±13.0	0.687
Functional capacity, n(%)			
NYHA class I	0	0	NA
NYHA class II	53（67.1%）	95（65.1%）	0.771
NYHA class III	17（21.5%）	33（22.6%）	0.869
NYHA class IV	9（11.4%）	18（12.3%）	1.000
Type of atrial fibrillation			
Paroxysmal, n(%)	22（27.8%）	43（29.5%）	0.878
Persistent, n(%)	57（72.2%）	103（70.5%）	0.878
Permanent, n(%)	0	0	NA
Chronic obstructive pulmonary disease, n(%)	0	1（0.68%）	1.000
Hypertension, n(%)	30（37.9%）	39（26.7%）	0.096
Palpitations, n(%)	37（46.8%）	57（39.0%）	0.322
Type II diabetes mellitus, n(%)	9（11.4%）	15（10.3%）	0.823
Pulmonary artery hypertension, n(%)	18（22.8%）	53（36.3%）	0.050
Disease of the extracardiac arterial system, n(%)	8（10.1%）	30（20.5%）	0.061
Left auricular thrombus, n(%)	0	2（1.37%）	0.542
Previous history of radiofrequency	0	1（0.68%）	1.000

ablation, n(%)			
Previous history of heart surgery, n(%)	2（2.53%）	10（6.85%）	0.287
Preoperative ECG, n(%)			
atrial fibrillation	70（93.3%）	128（87.7%）	1.000
atrial flutter	4（5.06%）	7（4.79%）	1.000
sinus rhythm	5（6.33%）	11（7.53%）	0.794
Preoperative cardiac ultrasonography, mean ± SD			
LAD（mm）	48.33±6.92	49.05±7.94	0.496
LASID（mm）	73.62±11.78	71.92±12.42	0.320
LAMD（mm）	58.0±13.0	59.0±16.0	0.978
MVG（mmHg）	5.40±3.50	5.60±2.64	0.823
LVDs（mm）	34.0±6.0	32.0±6.0	0.003
LVDd（mm）	51.0±9.0	49.0±10.5	0.089
LVEF（%）	60.68±5.77	61.61±7.10	0.321
Preoperative laboratory examination, mean ± SD			
uric acid	385.0±121.0	375.5±130.7	0.881
platelet	191.0±108.0	191.5±71.75	0.482
B-type natriuretic peptide	198.0±242.0	160.5±170.5	0.808
hypersensitive C-reactive protein	3.10±3.00	3.00±3.40	0.663
erythrocyte	5.00±5.00	6.00±5.00	0.080

sedimentation rate			
D-dimer	345.0±477.0	300.0±439.3	0.252
Euroscore, mean ± SD	4.00±4.00	5.00±4.00	0.730

Table S2: Characteristics and events during the operation

	mi-MAZE	os-MAZE	*P value*
Number	79	146	-
Surgical procedures, n (%)			
mitral vale replacement	55（69.6%）	120（82.2%）	0.043
mitral valve plasty	24（30.4%）	26（17.8%）	0.043
Tricuspid valve plasty	23（29.1%）	95（65.1%）	<0.001
Temporary pacemaker	58（73.4%）	78（53.4%）	0.004
Left auricle			
suture	65（82.3%）	50（34.2%）	<0.001
ligation	7（8.86%）	94（64.4%）	<0.001
excision	7（8.86%）	2（1.4%）	0.017
extracorporeal circulation, mean ± SD			
cardiopulmonary bypass (CPB) time(min)	109.32±19.0	99.69±25.6	0.005
aortic cross-clamping (ACC) time(min)	76.0±20.3	59.0±27.0	<0.001
operation time, mean ± SD,(min)	195.0±46.0	199.0±60.8	0.472
Intraoperative adverse events, n (%)			
Atrioventricular block	0	0	NA
Low cardiac output	0	0	NA
Myocardial infarction	0	0	NA

Acute renal insufficiency	0	0	NA
Death	0	0	NA
Conversion to open surgery	0	-	NA
At the end of surgery			
Sinus rhythm	76（96.2%）	135（92.5%）	0.412
Pacemaker rhythm	3（3.8%）	21（14.4%）	0.412

Table S3: The Prediction Model Simplified Scale A.

grade	risk assessment	n
0	None of the indicators are in the risk zone	23
1	Any one indicator	56
2	Any two indicators	47
3	≥3 indicators	42

Table S4: The Prediction Model Simplified Scale B.

grade	points	n
0	points=0	23
1	0<points≤5	34
2	5<points≤10	33
3	10<points≤15	32
4	15<points≤25	32
5	points≥25	14

Table S5: The Prediction Model Simplified Scale C.

grade	points	n
Low risk	0≤points≤5	57
Medium risk	5<points≤10	65
High risk	points≥15	46

Table S6: Validation set: univariate Cox regression analysis for BLUCE.

grade	n	recurrence(%)	HR（95%CI）	P value
BLUCE model				0.044
Low risk	11	1（9.09%）	1	
Medium risk	22	6（27.27%）	3.104（0.374-25.795）	0.294
High risk	23	12（52.17%）	6.637（0.862-51.091）	0.069

Code of R-language

```
install.packages("rms")

install.packages("openxlsx")


rm(list=ls())

library(openxlsx)

library(survival)

library(lattice)

library(Formula)

library(ggplot2)

library(Hmisc)

library(rms)

lxrdata <- read.xlsx("R168.xlsx", sheet=1)

dd<-datadist(lxrdata)

options(datadist="dd")

f <- cph(Surv(time,censored) ~ UA+BNP+CRP+ESR+LAD, x=T,y=T,surv=T, data=lxrdata,
time.inc=1)

surv <- Survival(f)

nom <- nomogram(f,fun=list(function(x) surv(3,x),function(x) surv(6, x), function(x) surv(12, x),
function(x) surv(36, x)),
```

```
lp=F, funlabel=c("3-month simus rhythm","6-month sinus rhythm", "1-year sinus rhythm", "3-
year sinus rhythm"),
maxscale=10, fun.at=c(0.95, 0.9, 0.85, 0.8, 0.75, 0.7, 0.6, 0.5,0.4,0.3,0.2,0.1))
plot(nom)
validate(f, method="boot", B=1000, dxy=T)
rcorrcens(Surv(time, censored) ~ predict(f), data = lxrdata)
cindex<-survConcordance(Surv(time,censored)~predict(f),data=lxrdata)
cindex$concordance
## internal verification
cal3<-calibrate(f,cmethod="KM",method="boot",u=3,m=50,B=1000)
plot(cal3,lwd=2,lty=1,  errbar.col=c(rgb(0,118,192,maxColorValue=255)),
     xlim=c(0,1),ylim=c(0,1),xlab="Nomogram-Predicted Probability of 3-month sinus rhythm",
     ylab="Actual 3-month sinus rhythm(proportion)",
     col=c(rgb(192,98,83,maxColorValue = 255)))
cal6<-calibrate(f,cmethod="KM",method="boot",u=6,m=50,B=1000)
plot(cal6,lwd=2,lty=1,  errbar.col=c(rgb(0,118,192,maxColorValue=255)),
     xlim=c(0,1),ylim=c(0,1),xlab="Nomogram-Predicted Probability of 6-month sinus rhythm",
     ylab="Actual 6-month sinus rhythm(proportion)",
     col=c(rgb(192,98,83,maxColorValue = 255)))
cal12<-calibrate(f,cmethod="KM",method="boot",u=12,m=50,B=1000)
plot(cal12,lwd=2,lty=1,  errbar.col=c(rgb(0,118,192,maxColorValue=255)),
     xlim=c(0,1),ylim=c(0,1),xlab="Nomogram-Predicted Probability of 1-year sinus rhythm",
     ylab="Actual 1-year sinus rhythm(proportion)",
     col=c(rgb(192,98,83,maxColorValue = 255)))
cal36<-calibrate(f,cmethod="KM",method="boot",u=36,m=50,B=1000)
plot(cal36,lwd=2,lty=1,  errbar.col=c(rgb(0,118,192,maxColorValue=255)),
     xlim=c(0,1),ylim=c(0,1),xlab="Nomogram-Predicted Probability of 3-year sinus rhythm",
     ylab="Actual 3-year sinus rhythm(proportion)",
     col=c(rgb(192,98,83,maxColorValue = 255)))
```

```
nom$UA$points
nom$BNP$points
nom$CRP$points
nom$ESR$points
nom$LAD$points

## external verification
yzdata <- read.xlsx("R56.xlsx", sheet=1)
f0 <- cph(Surv(time, censored) ~ BLUCE,x=T, y=T, surv=T, data=yzdata, time.inc=1)
validate(f0, method="boot", B=1000, dxy=T)
rcorrcens(Surv(time, censored) ~ predict(f0), data = yzdata)

cal03<-calibrate(f0,cmethod="KM",method="boot",u=3,m=10,B=1000)
plot(cal03,lwd=2,lty=1,    errbar.col=c(rgb(0,118,192,maxColorValue=255)),
        xlim=c(0,1),ylim=c(0,1),xlab="Nomogram-Predicted Probability of 3-month sinus rhythm",
        ylab="Actual 3-month sinus rhythm(proportion)",
        col=c(rgb(192,98,83,maxColorValue = 255)))
cal06<-calibrate(f0,cmethod="KM",method="boot",u=6,m=10,B=1000)
plot(cal06,lwd=2,lty=1,    errbar.col=c(rgb(0,118,192,maxColorValue=255)),
        xlim=c(0,1),ylim=c(0,1),xlab="Nomogram-Predicted Probability of 6-month sinus rhythm",
        ylab="Actual 6-month sinus rhythm(proportion)",
        col=c(rgb(192,98,83,maxColorValue = 255)))
cal012<-calibrate(f0,cmethod="KM",method="boot",u=12,m=10,B=1000)
plot(cal012,lwd=2,lty=1,    errbar.col=c(rgb(0,118,192,maxColorValue=255)),
        xlim=c(0,1),ylim=c(0,1),xlab="Nomogram-Predicted Probability of 1-year sinus rhythm",
        ylab="Actual 1-year sinus rhythm(proportion)",
        col=c(rgb(192,98,83,maxColorValue = 255)))
cal036<-calibrate(f0,cmethod="KM",method="boot",u=36,m=10,B=1000)
plot(cal036,lwd=2,lty=1,    errbar.col=c(rgb(0,118,192,maxColorValue=255)),
        xlim=c(0,1),ylim=c(0,1),xlab="Nomogram-Predicted Probability of 3-year sinus rhythm",
```

```
ylab="Actual 3-year sinus rhythm(proportion)",
col=c(rgb(192,98,83,maxColorValue = 255)))
```
